# Thymoquinone alleviates the accumulation of ROS and pyroptosis and promotes perforator skin flap survival through SIRT1/NF-κB pathway

**DOI:** 10.3389/fphar.2025.1567762

**Published:** 2025-03-25

**Authors:** Jianxin Yang, Haojie Zhang, Libin Ni, Jun He

**Affiliations:** ^1^ Department of Orthopaedics, Zhejiang Hospital, Hangzhou, Zhejiang, China; ^2^ Department of Spine Surgery, The Third Affiliated Hospital, Sun Yat-Sen University, Guangzhou, China

**Keywords:** perforator flap, thymoquinone, pyroptosis, SIRT1, NF-κB/NLRP3 signaling pathway

## Abstract

Perforator flap transplantation is an important technique in flap reconstructive surgery, but flap necrosis limits its clinical effectiveness. Thymoquinone (TQ), a natural bioactive plant quinone found in black seed, exhibits anti-inflammatory, angiogenic, and antimicrobial properties. This study investigates the therapeutic effects of TQ in a perforator flap model through *in vivo* and *in vitro* experiments. Human umbilical vein endothelial cells (HUVECs) were treated with Tert-butyl Hydroperoxide (TBHP) to simulate an *in vitro* flap model and were then treated with TQ. *In vivo* experiments used a rat perforator flap model, and vascularization was assessed using Doppler ultrasound on days 3 and 7 after flap creation. On day 7 post-surgery, flap samples were collected to evaluate vascularity, reactive oxygen species, apoptosis and pyroptosis. Network pharmacology analysis was conducted to identify relevant signaling pathways, and molecular docking techniques were used to predict potential target binding sites. *In vitro* results showed that both TQ treatment and NLRP3 inhibitors reduced the expression of pyroptosis-related proteins. *In vivo* results indicated that the TQ-treated group had increased flap survival area, blood flow intensity, and microvascular density, while oxidative stress, apoptosis, and pyroptosis levels were reduced. Angiogenesis was enhanced, and expression of the SIRT1 protein was increased, while the p-P65/NF-κB/NLRP3 pathway was downregulated. After treatment with a SIRT1 inhibitor, flap survival rate and angiogenesis were reduced. These findings suggest that TQ improves perforator flap survival by inhibiting the NF-κB/NLRP3 pathway and promoting angiogenesis.

## Introduction

For a considerable length of time, multi-regional perforator flaps have been widely utilized in clinical settings for the treatment of dermatological conditions, including congenital abnormalities, traumatic injuries, tumor ablations, and skin ulcers resulting from diabetes (Cheng et al., 2017). However, due to the limited blood supply range of skin arteries, insufficient blood perfusion often occurs in the flap area during the early stages, leading to ischemic injury in the distal portion of the flap (Peng et al., 2022). With neovascularization and vasodilation, reperfusion injury may further occur in the distal part of the flap (Liu et al., 2019a). Therefore, ischemia-reperfusion (I/R) injury is one of the primary factors contributing to distal flap necrosis, which limits its clinical application. I/R injury leads to the accumulation of reactive oxygen species (ROS), ultimately resulting in cell death. Both ROS and apoptosis can accumulate due to I/R injury, ultimately leading to cellular demise (Xie et al., 2023). Previous studies have demonstrated that inhibiting oxidative stress, apoptosis can reduce the risk of ischemia-reperfusion injury and distal necrosis in flaps (Jiang et al., 2022; Chen et al., 2022).

Sirtuin 1 (SIRT1), an NAD+-dependent deacetylase, is involved in regulating angiogenesis, inflammation, oxidative stress, and apoptosis, including the inhibition of NLRP3 inflammasomes (Chen et al., 2013). To date, SIRT1 has been found to attenuate damage resulting from cerebral I/R in rats (Mei et al., 2022). Furthermore, under injury conditions, SIRT1 can enhance cardiac and neuroprotective activities (Hu et al., 2022; Xu et al., 2021). Recent studies have also indicated that SIRT1 can aid in the survival of random-pattern flaps (Jiang et al., 2022). However, uncertainties remain regarding the mechanisms controlling SIRT1 in the survival of multi-regional perforator flaps.

Cell pyroptosis, a recently discovered form of programmed cell death accompanied by inflammation, is regulated by numerous signaling pathways, including the most frequently studied NLRP3-Caspase-1-GSDMD pathway (He et al., 2015). The NLRP3 inflammasome detects endogenous and microbial danger signals and initiates innate immune responses. Upon activation, cytosolic NLRP3 oligomerizes with other inflammasome complex proteins, particularly ASC and pro-caspase-1. Pro-caspase-1 is then activated, leading to the cleavage of GSDMD (Man and Kanneganti, 2016). Following activation, the N-terminal portion of GSDMD oligomerizes and generates membrane pores, causing cell swelling, plasma membrane rupture, and release of cytosolic contents (Wang et al., 2019). Previous studies have shown that pyroptosis is a crucial pathological event in various diseases (Wan et al., 2020; Zhaolin et al., 2019), especially in vascular diseases (Zhou et al., 2018). Tissue ischemia-reperfusion injury and the development of microvascular diseases are significant factors contributing to flap necrosis (Odake et al., 2021). Recent research has highlighted the role of pyroptosis in maintaining vascular homeostasis and cardiovascular diseases, with NLRP3 inflammasome-induced pyroptosis playing a key role in vascular endothelial dysfunction (Bergsbaken et al., 2009). As a specific inhibitor of NLRP3, MCC950 has recently been found to effectively inhibit pyroptosis in various diseases, such as inflammatory bowel disease, diabetic cardiomyopathy, kidney injury (Wang et al., 2024; Ba et al., 2025; Luo et al., 2025). Therefore, tailored modulation of pyroptosis may represent a novel approach to improving the survival of multi-regional perforator flaps, as pyroptosis may play a pivotal role in inflammation associated with ischemia-reperfusion injury.

Thymoquinone (TQ), a pharmacologically active plant quinone found in the seeds of black cumin, is used in folk medicine worldwide for the treatment and prevention of various diseases and disorders (Ahmad et al., 2016). Notably, TQ has been reported to possess a wide range of biological activities, including anti-inflammatory (Wang et al., 2015; Chen et al., 2016), antioxidant (Lu et al., 2018), anti-tumor (Darakhshan et al., 2015), and immunomodulatory effects (Salem, 2005). Additionally, TQ can promote burn wound healing by reducing inflammation and oxidative stress (Selçuk et al., 2013). Recently, several studies have demonstrated the protective effects of TQ on cardiac injury, such as reperfusion injury in the context of ischemic damage and acute abdominal aortic ischemia, mediated through the pyroptosis pathway (Liu et al., 2019b). Currently, there are few studies exploring whether TQ has the potential to inhibit pyroptosis and promote angiogenesis in perforator flap models. Therefore, we aim to investigate the effects of TQ on flap survival and explore the biological mechanisms in multi-regional perforator flaps.

## Materials and methods

### Animal details

Healthy male Sprague Dawley rats (250–300 g), aged 6–8 weeks, were provided by the Experimental Animal Center of Wenzhou Medical University (license no. SCXK [ZJ] 2015-0001). The animal research procedures adhered to the ethical standards established by the National Institutes of Health for animal experiments. The use of these rats in the current investigation was approved by the Animal Research Committee of Wenzhou Medical University (wydw2023-0003). The rats were housed in a sanitary environment with a controlled 12-h light/dark cycle at a temperature of 25°C and had access to food and water *ad libitum*. The rats were randomly assigned to one of three experimental groups: the control group (n = 25), the TQ (TQ) group (n = 25), and the TQ+EX527 group (n = 15).

### Reagents and antibodies

TQ (TQ; purity 99.48%) and MCC950 (purity 99.62%) are provided by MedChemExpress LLC (USA). EX527 is purchased from meilunbio (China). Sodium pentobarbital, hematoxylin and eosin staining kits, lead oxide-gelatin, and diaminobenzidine (DAB) are procured from Solarbio Life Science (China). Anti-cadherin 5 is provided by Wuhan Boster Biological Technology, Ltd. Primary antibodies against GAPDH, HO1, SOD1, Bax, Bcl-2, GSDMD-N, and VEGF are sourced from The Proteintech Group (Chicago, USA). Antibodies against caspase-3, NLRP3, IL-18, IL-1β, SIRT1, p-P65, P65, and IkB-α are obtained from Cell Signaling Technologies (Beverly, Massachusetts). The goat anti-rabbit IgG secondary antibody is provided by Santa Cruz Biotechnology Inc. (Dallas, Texas, USA). The ECL Plus Reagent kits and BCA Kits are obtained from PerkinElmer Life Sciences and Beyotime Biotechnology, respectively.

### Flap animal model

Rats are anesthetized using isoflurane. Subsequently, back hair is removed using an electric razor and depilatory cream. The remainder of the surgical procedure is performed in a sterile environment. The anatomical landmarks of the rats influence the size of the skin flaps. The longitudinal axis of the spine serves as the basis for the medial edge (posterior midline) of the skin flap. The lateral boundary is positioned 2.5 cm away from the medial boundary. The lateral and medial edges converge at the anterior iliac spine, forming the caudal edge, which acts as an external barrier. As previously mentioned, the skin flap measures approximately 2.5 × 11 cm. Once the skin flap is fully elevated from the underlying fascia, hemostasis is achieved satisfactorily. The flap model comprises three vascular territories: the anatomic territory, represented by the deep circumflex iliac (DCI) vascular plexus, the dynamic territory, represented by the intercostal (IC) vascular plexus, and the potential territory, represented by the thoracodorsal (TD) vascular plexus. The DCI vascular plexus is preserved, while the other two vascular plexuses are ligated. The skin flap is then sutured in place using 4–0 silk. On postoperative day 7, all rats are euthanized, and the skin flap tissues are collected for further investigation.

### Cell culture

As previously reported in studies aiming to simulate apoptosis and oxidative stress injury *in vitro* experiments involving skin flaps, Human Umbilical Vein Endothelial Cells (HUVECs) were selected (Lu et al., 2024). HUVECs were obtained from the American Type Culture Collection (ATCC; Manassas, VA) and cultured in Dulbecco’s Modified Eagle Medium (DMEM; Gibco, Carlsbad, USA), supplemented with 10% Fetal Bovine Serum (FBS; Gibco, Carlsbad, USA) and 1% Penicillin-Streptomycin (Gibco, Carlsbad, USA). The cells were maintained in a humidified incubator at 37°C with 5% CO2. Upon reaching 70%–80% confluence, HUVECs were subjected to the corresponding treatments and utilized for subsequent experimental procedures.

### Drug treatment

For *in vitro* experiments: The Tert-butyl Hydroperoxide (TBHP) group was incubated with 100 μM TBHP for 24 h, the selection of concentrations is referenced to previous literature (Chen et al., 2022). The TBHP + TQ (TQ) group was simultaneously incubated with 100 μM TBHP and 10 μM TQ for 24 h. The TBHP + MCC950 group was pretreated with 10 μM MCC950 for 30 min prior to the addition of 100 μM TBHP and then incubated for 24 h. For *in vivo* experiments, the TQ group received daily gavage of 2 mg/kg TQ for 7 days post-surgery, while the control group received an equal volume of dimethyl sulfoxide (DMSO). The TQ + EX527 group received an intraperitoneal injection of EX527 (10 mg/kg/day) 30 min prior to TQ administration.

### Measurement of perforator flap survival area

Within 7 days after establishing the perforator flap model, the appearance of the flaps was observed. On postoperative days 3 and 7, five rats from each group were randomly selected and anesthetized. High-resolution photographs of the dorsal flaps were taken using a digital camera to measure the percentage of survival area as follows: (survival flap area/total flap area) × 100%.

### Doppler blood flow measurement

Doppler blood flow measurement was used to detect blood flow in the flaps of live rats. On postoperative days 3 and 7, five rats from each group were randomly selected and anesthetized. The flaps were scanned using a Doppler instrument in a stable state. To assess blood flow in the flaps, the images were imported into moorLDI Review software for quantitative analysis. Each rat was tested three times, and the average values were calculated for statistical analysis.

### Hematoxylin and eosin staining

On the seventh postoperative day (POD), three tissue samples (0.5 cm × 0.5 cm) were collected from the middle of the surgical critical zone in each group for histopathological analysis. After perfusing with phosphate-buffered saline (PBS) solution to remove blood cells, the specimens were fixed in 4% paraformaldehyde for 24 h and subsequently embedded in paraffin. The paraffin-embedded samples were then sectioned transversely into 4-μm-thick slices. These slices were stained with hematoxylin and eosin (H&E) according to standard histological protocols provided by Solarbio Science and Technology (Beijing, China; Catalog No. G1120). Six randomly selected regions in three random sections from each sample were observed under a light microscope (Olympus Corp, Tokyo, Japan). Additionally, to assess the microcirculation level, the number of microvessels per unit area (per square millimeter) in a random visual field was counted as a measure of microvascular density (MVD). This provided a quantitative evaluation of the microvasculature within the tissue samples.

### Immunohistochemical

The slides were first deparaffinized and rehydrated through a series of graded alcohols. After blocking endogenous peroxidase activity, the slides were incubated with primary antibodies against CD34 (1:100), cleaved caspase-3 (C-CASP3; 1:200), and superoxide dismutase 1 (SOD1; 1:100) overnight at 4°C. Following three washes, the slides were incubated with a secondary antibody for 1 h at 37°C. Images were then captured under an optical microscope and analyzed using Image Pro-plus software. The next day, the sections were soaked in PBS solution at room temperature for 5 min, with this step repeated three times. The sections were then incubated with horseradish peroxidase-labeled secondary antibody at 37°C for 30 min. In a darkroom, DAB (3,3′-diaminobenzidine) chromogenic solution was added dropwise to the tissues and observed under a microscope for 1–2 min, followed by rinsing with PBS solution. The nuclei were then stained with hematoxylin for 1 min and rinsed under slow running water to restore the blue color. Subsequently, the sections underwent gradient alcohol dehydration, xylene permeabilization, and were mounted with gum. Observations and recordings were made under a 200x optical microscope. In the later stage, specialized software was utilized to automatically analyze the absorbance of CD34, C-CASP3, and SOD1, as well as to count the number of blood vessels with positive expression of CD34. This provided a quantitative assessment of the immunohistochemical staining results.

### Immunofluorescence staining

For *In Vitro* Studies: HUVECs are seeded onto glass dishes and treated with the corresponding designated reagents. After fixation, the cells are permeabilized with Triton X-100 for 10 min at room temperature. Subsequently, the cells are blocked with goat serum for 1 h and incubated with primary antibodies against GSDMD-N (1:200), Caspase-1 (1:200), and NLRP3 (1:200) overnight at 4°C. Secondary antibodies are then added and incubated at 37°C for 1 h. The cell nuclei are stained with DAPI solution. Images are captured using a fluorescence microscope, and the integrated optical density is analyzed using software. For *In Vivo* Studies: Following immunohistochemical procedures, six samples undergo dewaxing and rehydration. After antigen retrieval and blocking, primary antibodies diluted in 10% goat serum in PBS (GSDMD-N 1:100, SIRT1 1:100, VEGF 1:100) are applied and incubated overnight at 4°C. Subsequently, FITC-labeled goat anti-rabbit IgG secondary antibody (1:200) is incubated at room temperature for 1 h. The sections are soaked in PBS for 5 min, repeated three times. An appropriate amount of DAPI solution is added to the slides for 5 min, and observations are made under a 200x fluorescence microscope. Later, software is used to count the number of GSDMD-N, SIRT1, and VEGF positive cells.

### Western blotting

Protein extracts from animal samples and total cellular proteins isolated from HUVECs were obtained using RIPA lysis buffer supplemented with 1 mM PMSF. Subsequently, protein concentrations were determined using a BCA kit sourced from Beyotime Biotechnology in China. The proteins were then subjected to electrophoresis and transferred onto PVDF membranes manufactured by Millipore, located in Bedford, MA, USA. Following blocking with NcmBlot blocking buffer, provided by New Cells and Molecular Biotech in China, the membranes were incubated with their respective antibodies overnight at a temperature of 4°C. Afterward, the membranes were further incubated with secondary antibodies for a duration of 2 h. Finally, the grayscale values of the blots were quantitated and analyzed using Image Lab 3.0 software, developed by Bio-Rad in California, United States.

### Collection of targets for ischemia reperfusion injury and prediction of thymoquinone targets

To identify disease targets related to the perforator flap, we conducted a database search using keywords such as ‘ischemia reperfusion injury’ and found relevant targets in GeneCards. Additionally, we obtained targets for TQ using SwissTargetPrediction with a canonical SMILE downloaded from PubChem. These target genes were then intersected for further KEGG analysis.

### KEGG enrichment analysis

We utilized Metascape (version 3.5.20230101) to perform KEGG enrichment analysis on the intersection target genes. Metascape employs the hypergeometric test and Benjamini–Hochberg p-value correction algorithm to identify ontology terms with a statistically significant number of genes in common with the input list. KEGG serves as a comprehensive knowledge base for analyzing gene functions and related pathways.

### Molecular docking

The SIRT1 protein’s three-dimensional model was constructed using the SWISS-MODEL online tool with a template PDB ID of 5BTR, revealing a 98.44% homology between the target protein and its template. The TQ ligand’s molecular structure was downloaded from PubChem. PyMOL 2.3.0 was used to remove water molecules and original ligands from the downloaded target protein, while Chem3D software (version 2020) optimized the molecular mechanics of the preferred conformation of the small molecule. AutoDock Tools 1.5.6 prepared the pdbqt files for docking simulations, which were conducted using AutoDock Vina v.1.2.0 with the Lamarckian Genetic Algorithm and semi-flexible docking. The exhaustiveness parameter was set to 8, and the maximum number of output conformations was set to 9. PyMOL 2.3.0 was used for 3D visualization, while Discovery Studio was employed for 2D visualization of the docking complex.

### Statistical analysis

All experiments were executed in a randomized and double - blinded manner by investigators who were masked to the experimental groups. The results are presented as mean ± SEM, with the group size (n) indicated for each experimental group/condition, representing independent values rather than replicates. To minimize unwanted variability, the entire dataset was normalized. Statistical analysis was performed using SPSS version 19 (Chicago, IL, United States). Independent-sample t tests were employed to determine remarkably significant differences between two groups. Statistical analysis between multiple groups was performed by one-way ANOVA or Tukey’s multiple comparison test. Significance was determined at p < 0.05. Additionally, KEGG pathway analysis results with a p value <0.05 were considered statistically significant.

## Results

### TQ enhanced the survival rate and angiogenesis in rat perforator flaps following ischemia-reperfusion injury

**FIGURE 1:**
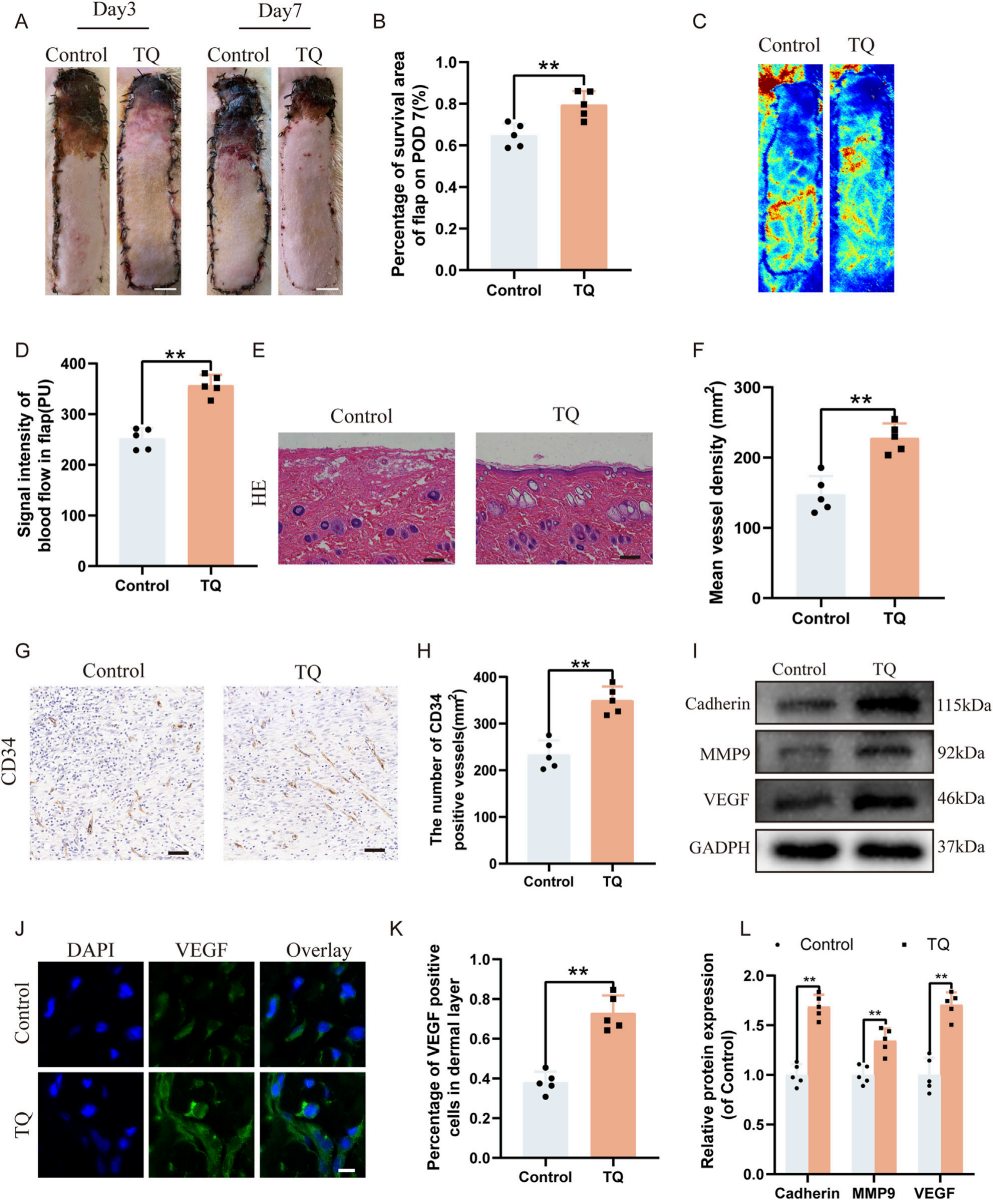
TQ enhances the survival rate and angiogenesis of multi-regional perforator flaps. **(A)** Gross images of dorsal flaps in the control and TQ groups on postoperative days 3 and 7. (scale bar: 1 cm) **(B)** Histogram showing the percentage of flap survival area in each group on postoperative day 7. **(C)** Doppler imaging of blood supply in perforator flaps of the control and TQ groups. **(D)** Quantitative assessment of blood flow intensity in flaps of the control and TQ groups. **(E)** H&E staining of flap tissues in the control and TQ groups (scale bar, 50 μm). **(F)** Quantitative assessment of microvascular density (MVD) in the control and TQ groups. **(G)** Immunohistochemical staining images of CD34 in the control and TQ groups (scale bar, 50 μm). **(H)** Quantitative assessment of CD34-positive vessel counts in the control and TQ groups. **(I)** Western blotting to detect the expression of angiogenesis-related proteins: Cadherin-5, MMP9, and VEGF in flap tissues of the control and TQ groups. **(J)** VEGF protein expression levels in flap tissues of rats in the control and TQ groups measured by immunofluorescence (scale bar: 20 µm). **(K)** Fluorescence intensity of VEGF expression analyzed using Image J. **(L)** Quantitative analysis of angiogenesis-related protein blots. Significance: *p < 0.05 and **p < 0.01. Data are presented as mean ± SEM, with five rats per group.

After establishing the perforator flap model, the survival and necrosis of the flaps in rats were observed and recorded. Over time, the boundary between survival and necrosis progressed from the distal end of the flap towards the pedicle, stabilizing by the seventh postoperative day in rats, with the distal areas of the flaps turning black, dry, and stiff (Figure 1A). Furthermore, we found that compared to the experimental control group, TQ significantly increased the survival area of the rat flaps (Figure 1B). Laser Doppler imaging revealed better blood perfusion in the TQ group, with significantly higher blood flow signal intensity in this group as well (Figures 1C, D). H&E staining showed a greater microvessel density in the TQ group (Figures 1E, F). Immunohistochemical staining data indicated an increase in CD34-positive vessel counts in the TQ group (Figures 1G, H). Based on these findings, we concluded that TQ improved the survival rate of rat perforator flaps following ischemia-reperfusion injury. Adequate blood flow and angiogenesis are among the most critical factors for perforator flap survival. To further determine whether TQ promotes angiogenesis after ischemia-reperfusion injury in perforator flaps, we used immunofluorescence and Western blot analysis to detect the expression of angiogenesis markers. Angiogenesis-related markers, including Cadherin-5, VEGF, and MMP9, were detected. Western blot results demonstrated that TQ treatment increased the levels of Cadherin-5, MMP9, and VEGF (Figures 1I, L). Additionally, immunofluorescence staining data showed a significant increase in VEGF-positive cell rates in the treatment group compared to the control group (Figures 1J, K). These results collectively indicate that TQ effectively promoted angiogenesis in perforator flaps, improving flap survival rates.

### TQ inhibits pyroptosis after ischemia-reperfusion injury in rat perforator flaps

**FIGURE 2:**
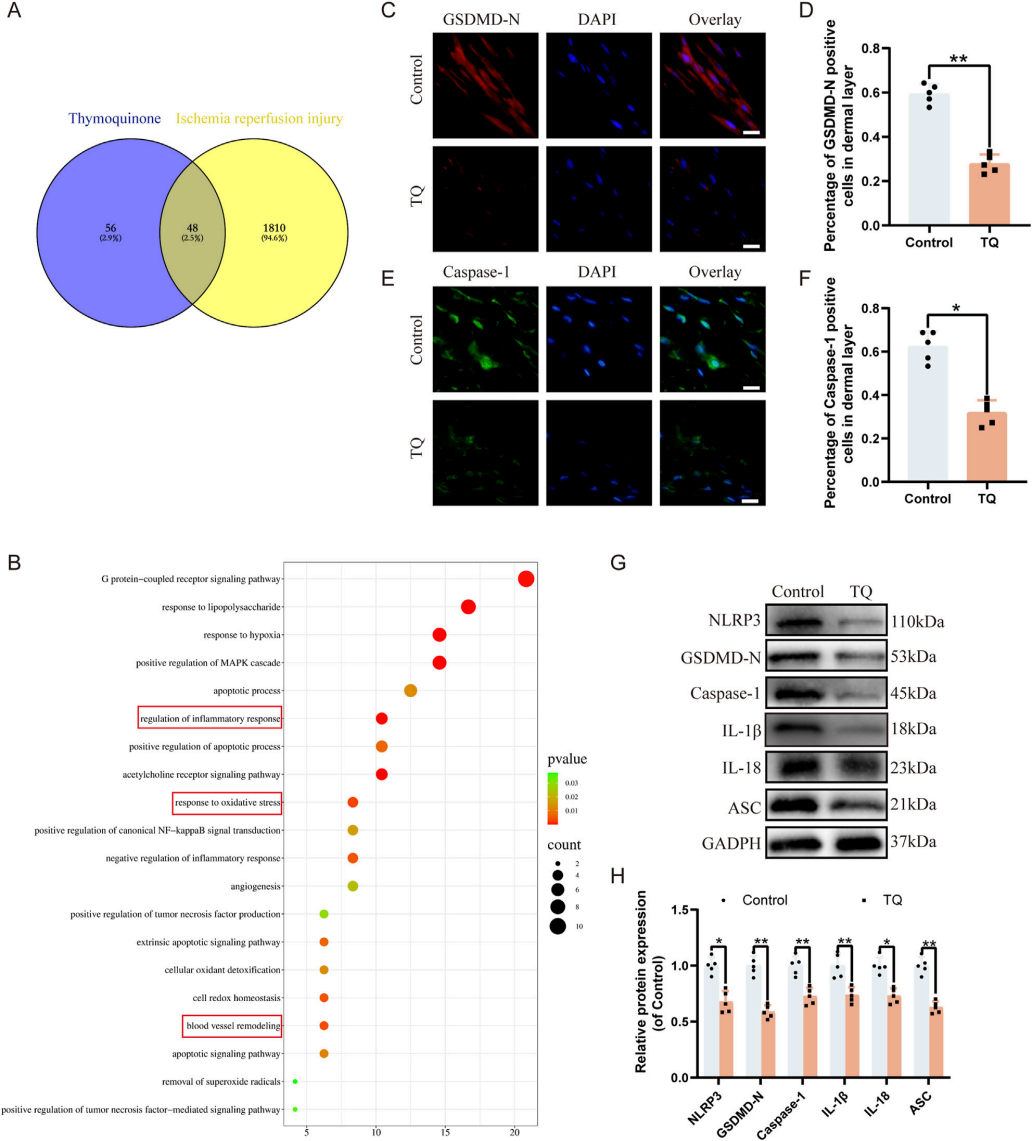
TQ inhibits pyroptosis in rat perforator flaps after ischemia-reperfusion injury. **(A)** Venn diagram showing overlapping targets of ischemia-reperfusion-related genes from GeneCards and predicted targets of TQ. **(B)** Top 15 enriched KEGG pathways for overlapping genes in **(A)**. **(C)** GSDMD-N protein expression levels in flap tissues of rats in the control and TQ groups measured by immunofluorescence (scale bar: 20 µm). **(D)** Fluorescence intensity of GSDMD-N expression analyzed using Image J. **(E)** Caspase-1 protein expression levels in flap tissues of rats in the control and TQ groups measured by immunofluorescence (scale bar: 20 µm). **(F)** Fluorescence intensity of Caspase-1 expression analyzed using Image J. **(G)** Western blotting to detect the expression of pyroptosis-related proteins: NLRP3, GSDMD-N, Caspase-1, IL-1β, IL-18, and ASC in flap tissues of the control and TQ groups. **(H)** Quantitative analysis of pyroptosis-related protein blots. Significance: *p < 0.05 and **p < 0.01. Data are presented as mean ± SEM, with five rats per group.

To determine which pathways are involved in the angiogenic effects of TQ, we searched for downstream targets of TQ and ischemia-reperfusion injury-related genes using SwissTargetPrediction and GeneCards. We took the intersection of these results, and a Venn diagram showed that 48 genes might be related to the therapeutic effects of TQ in ischemia-reperfusion injury (Figure 2A). Next, we selected these 48 genes for KEGG analysis, which revealed that the top 20 pathways were involved in the therapeutic effects of TQ, such as regulating inflammatory responses, oxidative stress, and apoptosis (Figure 2B). Inflammasome assembly of inflammatory mediators is an important manifestation of pyroptosis (Newton et al., 2024). It has been reported that pyroptosis is crucial for the development of ischemia-reperfusion injury, and inhibiting pyroptosis can effectively alleviate the progression of ischemia-reperfusion diseases (Qu et al., 2024; Chen et al., 2024). Based on the results of the KEGG enriched pathways, we speculated that the positive effects of TQ partly depend on the inhibition of pyroptosis. Using immunofluorescence staining, we assessed the levels of pyroptosis-related markers GSDMD-N and Caspase-1 to determine whether TQ leads to a reduction in pyroptosis in rat perforator flaps (Figures 2C–F). The analysis results indicated that TQ significantly decreased the percentage of GSDMD-N-positive and Caspase-1-positive cells in the dermis. Furthermore, Western blot analysis of pyroptosis-related proteins NLRP3, GSDMD-N, Caspase-1, IL-1β, IL-18, and ASC showed lower expression levels of these proteins in the TQ group compared to the control group (Figures 2G, H). To further explore the effects of TQ on pyroptosis, we conducted *in vitro* experiments using HUVECs. According to the immunofluorescence results, the expression levels of pyroptosis-related proteins GSDMD-N and Caspase-1 were significantly increased under TBHP stimulation but decreased under the action of TQ (Supplementary Figures S1A–D). Additionally, Western blot analysis showed that TBHP significantly increased the protein expression levels of NLRP3, GSDMD-N, Caspase-1, IL-1β, IL-18, and ASC, while TQ decreased their expression levels (Supplementary Figures S1E, F). To confirm our hypothesis, we conducted experiments using an NLRP3 inhibitor (Babuta et al., 2024). According to the immunofluorescence results, the expression level of NLRP3 was significantly decreased under the action of MCC950 (Supplementary Figures S1G, H). Moreover, Western blot analysis showed that the levels of pyroptosis-related proteins in the MCC950 group were also significantly inhibited (Supplementary Figures S1I, J). This indicates that MCC950 treatment significantly inhibited TBHP-induced pyroptosis. In summary, our results suggest that TQ inhibits pyroptosis in rat perforator flaps following ischemia-reperfusion injury.

### TQ ameliorates oxidative stress and apoptosis following ischemia-reperfusion injury in rat perforator flaps

**FIGURE 3:**
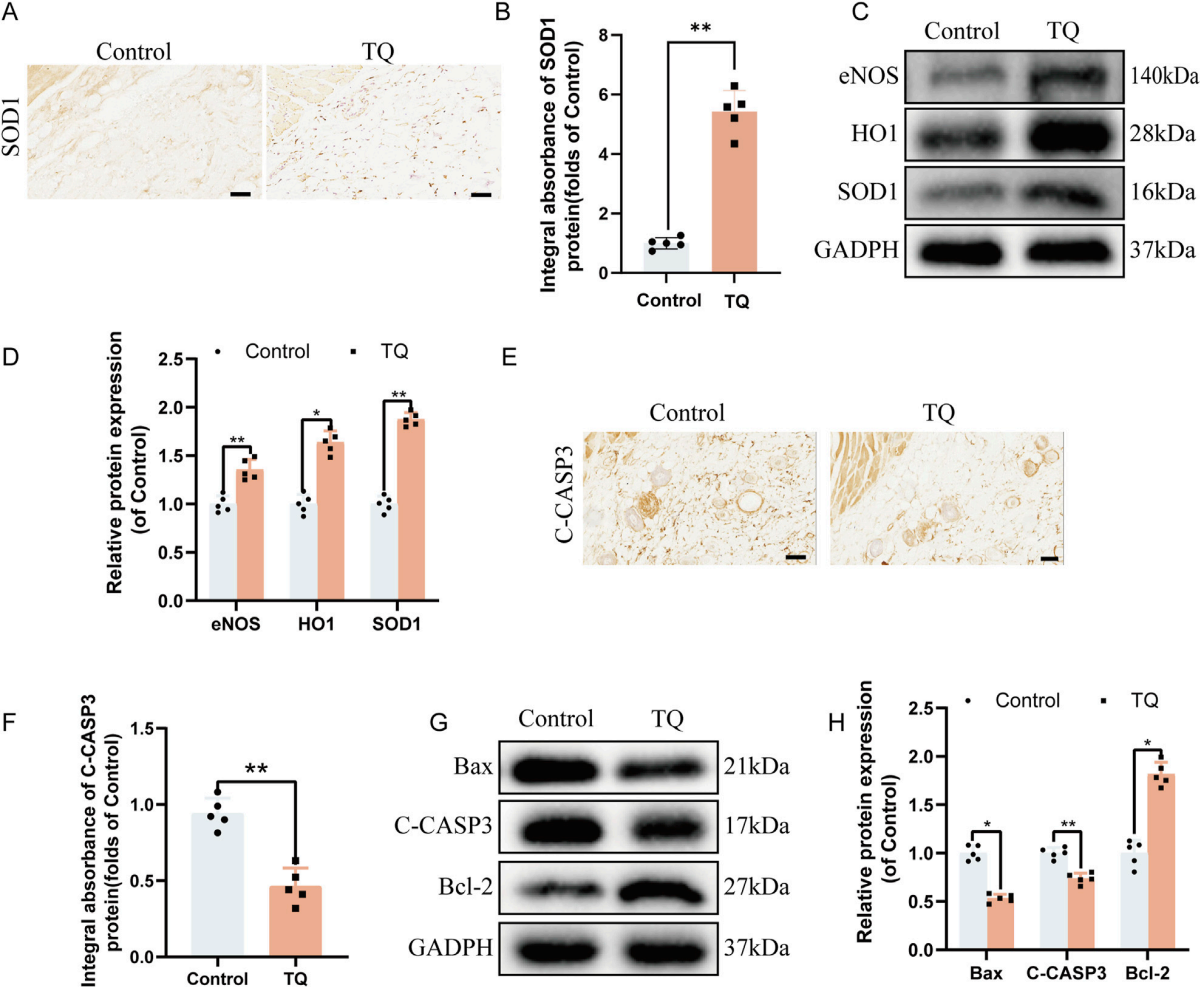
TQ Ameliorates Oxidative Stress and Apoptosis in Rat Perforator Flaps After Ischemia-Reperfusion Injury. **(A)** Immunohistochemical staining images of SOD1 in the control and TQ groups (scale bar, 50 μm). **(B)** Quantitative analysis of SOD1 absorbance in the control and TQ groups. **(C)** Western blotting to detect the expression of oxidative stress-related proteins: eNOS, HO1, and SOD1 in flap tissues of the control and TQ groups. **(D)** Quantitative analysis of oxidative stress-related protein blots. **(E)** Immunohistochemical staining images of C-CASP3 in the control and TQ groups (scale bar, 50 μm). **(F)** Quantitative analysis of C-CASP3 absorbance in the control and TQ groups. **(G)** Western blotting to detect the expression of apoptosis-related proteins: Bax, C-CASP3, and Bcl-2 in flap tissues of the control and TQ groups. **(H)** Quantitative analysis of apoptosis-related protein blots. Significance: *p < 0.05 and **p < 0.01. Data are presented as mean ± SEM, with five rats per group.

Oxidative stress is also crucial for the survival of perforator flaps. Immunohistochemical staining was employed to detect the expression of SOD1, an essential endogenous antioxidant enzyme (Nam et al., 2024), to further assess whether TQ can reduce oxidative stress following ischemia-reperfusion injury in rat perforator flaps. Immunohistochemical staining revealed that the SOD1 absorbance in the TQ group was higher than that in the control group (Figures 3A, B). Additionally, Western blot analysis was used to detect the expression levels of angiogenesis-related proteins, including eNOS, HO1, and SOD1, in multi-regional perforator flaps (Figures 3C, D). TQ treatment promoted the expression of these angiogenesis-related proteins. Collectively, these results suggest that TQ can ameliorate oxidative stress following ischemia-reperfusion injury in rat perforator flaps, which contributes to flap survival.

To investigate how TQ prevents necrosis in perforator flaps, the effects of apoptosis-related proteins on perforator flaps were assessed. According to immunohistochemical staining results, the C-CASP3 absorbance in the TQ group was lower compared to the control group (Figures 3E, F). Western blot results were consistent with immunohistochemical findings in terms of the expression of apoptosis-related proteins Bax and C-CASP3. The protein expression levels of Bax and C-CASP3 were significantly reduced, while the Bcl-2 protein expression level was significantly increased (Figures 3G, H). In summary, these findings indicate that a portion of the beneficial effects of TQ on the survival of rat perforator flaps following ischemia-reperfusion injury is attributed to the inhibition of apoptosis.

### The regulation of flap survival by TQ is related to the expression of SIRT1

Recent studies have also shown that SIRT1 can aid in the survival of random flaps (Jiang et al., 2022). We hypothesize that TQ may limit pyroptosis in rat perforator flaps through SIRT1 regulation, thereby reducing flap necrosis. We employed molecular docking analysis to explore the protective effects of TQ on flaps via the SIRT1/p65 pathway. As shown in Supplementary Table S1A, the binding affinity between the small molecule TQ and the target protein SIRT1 is −6.3 kcal/mol. Generally, ligands and receptor proteins can bind spontaneously if the binding energy is less than 0 kcal/mol, and they can bind stably if the binding energy is less than −4 kcal/mol^36^. A binding energy of less than −6 kcal/mol indicates a strong binding interaction. Therefore, a strong binding exists between TQ and SIRT1 protein.

**FIGURE 4:**
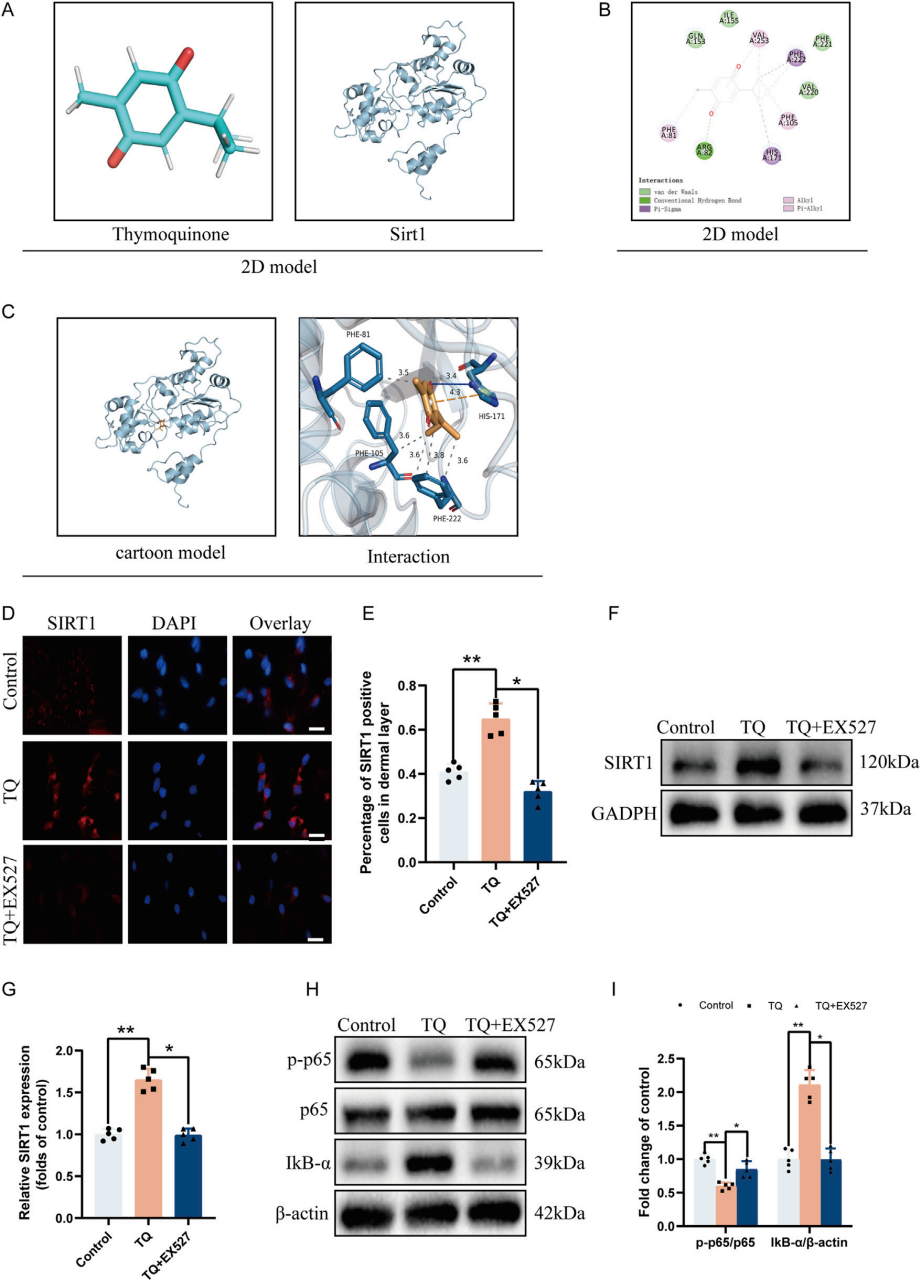
TQ Ameliorates Apoptosis in Rat Perforator Flaps After Ischemia-Reperfusion Injury by Targeting SIRT1. **(A)** Two-dimensional structural diagrams of TQ and SIRT1 protein. **(B)** Two-dimensional interaction diagram of the target protein with the ligand. **(C)** Overall view of TQ in the SIRT1 domain based on space-filling and ribbon models. **(D)** SIRT1 protein expression levels in flap tissues of rats in the control, TQ, and TQ+EX527 groups measured by immunofluorescence (scale bar: 20 µm). **(E)** Fluorescence intensity of SIRT1 expression in each group analyzed using Image J. **(F)** Western blotting to detect SIRT1 target protein expression in flap tissues of the control, TQ, and TQ+EX527 groups. **(G)** Quantitative analysis of SIRT1 protein blots. **(H)** Western blotting to detect the expression levels of NF-κB signaling pathway-related proteins in flap tissues of the control, TQ, and TQ+EX527 groups. **(I)** Quantitative analysis of NF-κB signaling pathway-related protein blots. Significance: *p < 0.05 and **p < 0.01. Data are presented as mean ± SEM, with five rats per group.

As illustrated in Figure 4C, the small ligand molecule binds to the “aromatic cage” of the target protein, with the interacting amino acids all being aromatic. These amino acids bind to phenylalanine (PHE) amino acids at positions 81, 105, and 22 of the receptor protein through strong hydrophobic interactions at distances of 3.5, 3.6, 3.6, 3.8, and 3.6 Å, respectively. Additionally, the ligand TQ binds to histidine (HIS) amino acid at position 171 of the receptor protein through a hydrogen bond with a distance of 3.4 Å. Simultaneously, HIS-171 also forms a π-cation interaction with the ligand. Furthermore, the two-dimensional interaction diagram (Figures 4A, B) analysis shows that, besides results similar to the three-dimensional interaction diagram (Figure 4C), there are van der Waals forces between the ligand molecule and multiple amino acids of the receptor protein. Due to the existence of these forces, the ligand small molecule can stably bind to the receptor protein. This indicates that the binding site between the ligand and the receptor protein has excellent binding free energy and good affinity. Therefore, the binding of TQ to SIRT1 protein is likely to exert the corresponding pharmacological effect.

To further confirm the binding of TQ to SIRT1 protein, we used the SIRT1 inhibitor EX527 (Li et al., 2023) before treating rat flaps with TQ. According to immunofluorescence, the number of SIRT1-positive cell expressions increased in the perforator flaps after TQ treatment (Figures 4D, E), while EX527 reversed the upregulation effect of TQ on SIRT1. Similarly, Western blot analysis revealed that TQ administration restored SIRT1 expression levels (Figures 4F, G), while EX527 attenuated the upregulation effect of TQ on SIRT1. Compared with the control group, the phosphorylated NF-κB p65 subunit decreased and IκB-α increased in the TQ group (Figures 4H, I), while EX527 reversed the dephosphorylation effect of TQ on p65 protein. These results suggest that TQ increases the expression of SIRT1 and decreases the expression level of phosphorylated NF-κB p65 after ischemia-reperfusion injury in rat perforator flaps.

### EX527 reverses the inhibitory effects of TQ on pyroptosis, oxidative stress, and apoptosis in rat perforator flaps following ischemia-reperfusion injury

**FIGURE 5:**
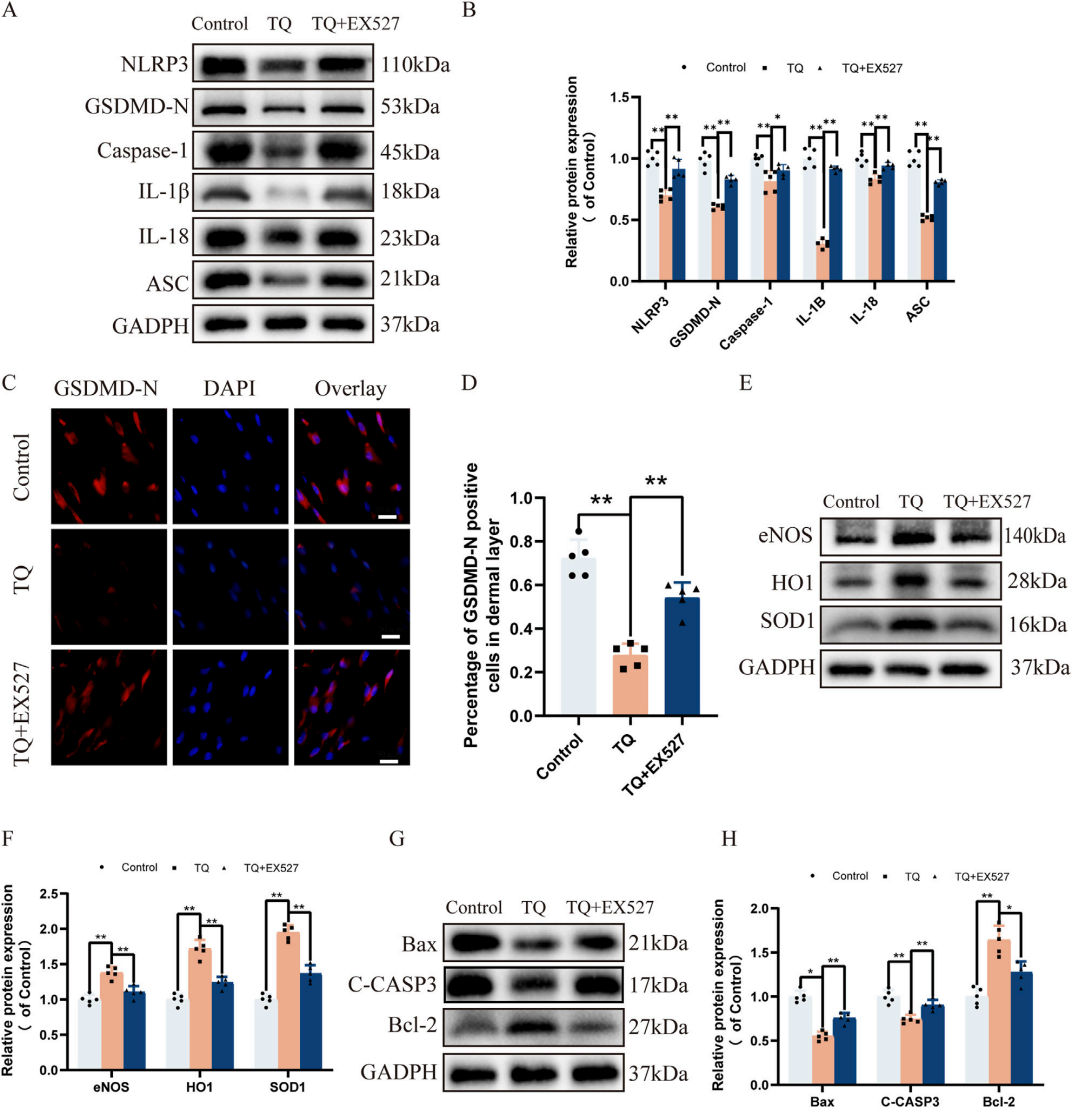
EX527 Reverses the Inhibitory Effects of TQ on Pyroptosis, Oxidative Stress, and Apoptosis in Rat Perforator Flaps After Ischemia-Reperfusion Injury. **(A)** Western blotting to detect the expression of pyroptosis-related proteins: NLRP3, GSDMD-N, Caspase-1, IL-1β, IL-18, and ASC in flap tissues of the control, TQ, and TQ+EX527 groups. **(B)** Quantitative analysis of pyroptosis-related protein blots. **(C)** GSDMD-N protein expression levels in flap tissues of rats in the control, TQ, and TQ+EX527 groups measured by immunofluorescence (scale bar: 20 µm). **(D)** Fluorescence intensity of GSDMD-N expression in each group analyzed using Image J. **(E)** Western blotting to detect the expression of oxidative stress-related proteins: eNOS, HO1, and SOD1 in flap tissues of the control, TQ, and TQ+EX527 groups. **(F)** Quantitative analysis of oxidative stress-related protein blots. **(G)** Western blotting to detect the expression of apoptosis-related proteins: Bax, C-CASP3, and Bcl-2 in flap tissues of the control, TQ, and TQ+EX527 groups. **(H)** Quantitative analysis of apoptosis-related protein blots. Significance: *p < 0.05 and **p < 0.01. Data are presented as mean ± SEM, with five rats per group.

Previous research has shown that the activation of SIRT1 target protein can inhibit NLRP3 inflammasome activation and subsequent caspase-1 cleavage and IL-1β secretion (Li et al., 2017). To investigate the impact of SIRT1 target protein on rat perforator flaps after ischemia-reperfusion injury, we used SIRT1 inhibitor EX527 prior to TQ treatment. Western blot analysis revealed that SIRT1 protein expression increased after TQ treatment, while the use of SIRT1 inhibitor EX527 increased the protein expression levels of NLRP3, GSDMD-N, Caspase-1, IL-1β, IL-18, and ASC (Figures 5A, B). Furthermore, for the pyroptosis marker protein GSDMD-N, its expression was inhibited after TQ treatment but increased after SIRT1 inhibitor treatment (Figures 5C, D). These findings suggest that SIRT1 inhibitor diminishes the ability of TQ to inhibit pyroptosis. We also investigated whether SIRT1 inhibitor could suppress the beneficial effects of TQ on oxidative stress and apoptosis in rat perforator flaps. Compared to the TQ group, SIRT1 inhibitor EX527 treatment significantly decreased the expression of eNOS, HO1, and SOD1 (Figures 5E, F). SIRT1 inhibitor EX527 treatment increased the expression of Bax and C-CASP3 and inhibited the expression of Bcl-2 (Figures 5G, H). Overall, these results indicate that the effects of TQ on pyroptosis, oxidative stress, and apoptosis in perforator flaps after ischemia-reperfusion injury may be associated with SIRT1.

### EX527 reverses the promotional effects of TQ on angiogenesis and survival of rat perforator flaps post ischemia-reperfusion injury

**FIGURE 6:**
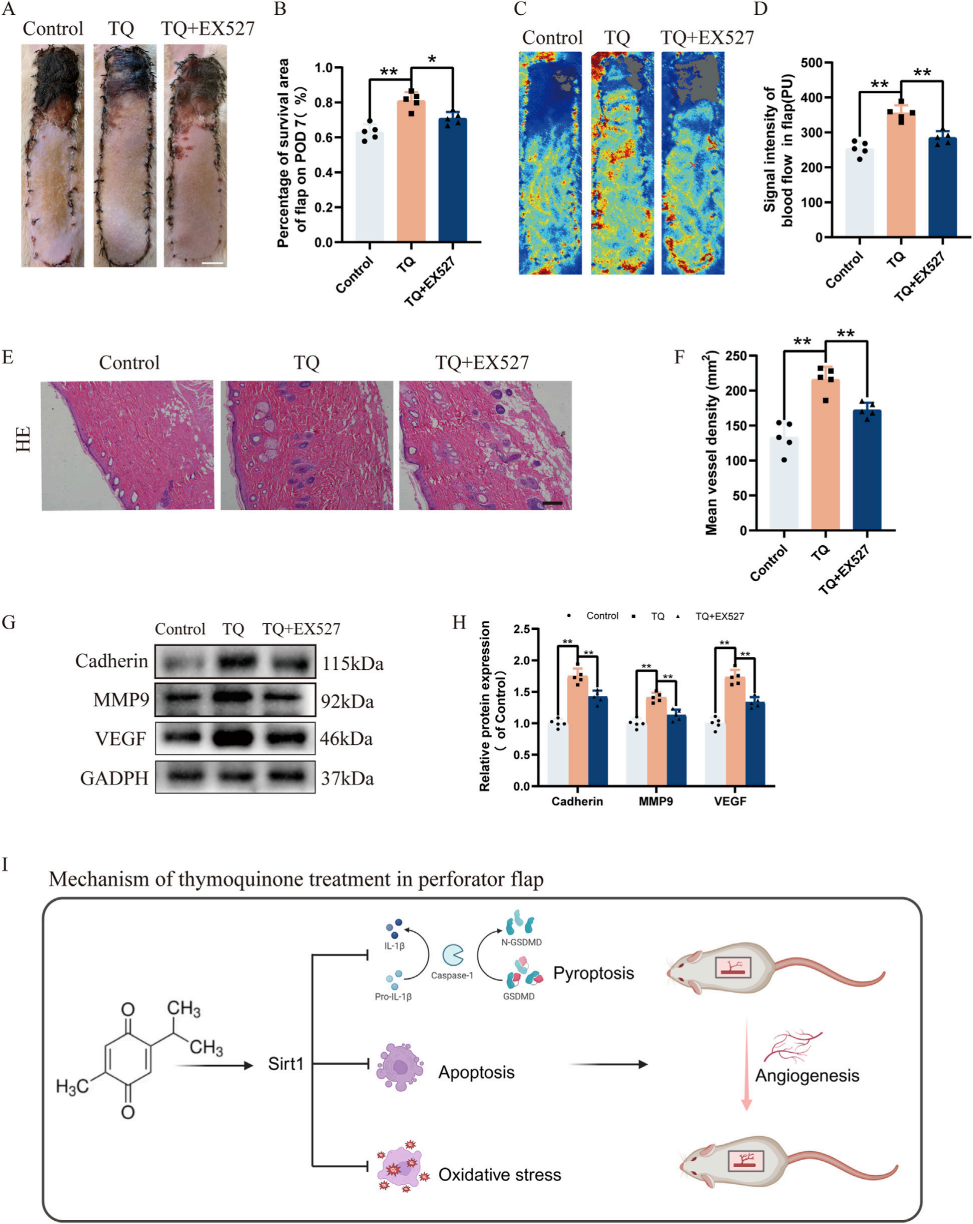
EX527 Reverses the Promoting Effects of TQ on Angiogenesis and Flap Survival in Rat Perforator Flaps After Ischemia-Reperfusion Injury. **(A)** Gross images of the dorsal flaps on the seventh postoperative day in the control, TQ, and TQ+EX527 groups. (scale bar, 1 cm) **(B)** A histogram showing the percentage of flap survival area on the seventh day for each group. **(C)** Doppler imaging of the blood supply in the perforator flaps of the control, TQ, and TQ+EX527 groups. **(D)** Quantitative examination of blood flow intensity in the flaps of each group. **(E)** H&E staining of flap tissues in the control, TQ, and TQ+EX527 groups (scale bar, 50 μm). **(F)** Quantitative examination of microvascular density (MVD) in each group. **(G)** Western blotting to detect the expression of angiogenesis-related proteins: Cadherin-5, MMP9, and VEGF in flap tissues of the control, TQ, and TQ+EX527 groups. **(H)** Quantitative analysis of angiogenesis-related protein blots. **(I)** Schematic diagram illustrating the protective effects of TQ on rat perforator flaps after ischemia-reperfusion injury. Significance: *p < 0.05 and **p < 0.01. Data are presented as mean ± SEM, with five rats per group.

To further confirm whether the promotional effects of TQ on angiogenesis and survival of rat perforator flaps subjected to ischemia-reperfusion injury are SIRT1-mediated, SIRT1 inhibitor EX527 was administered prior to TQ treatment. Compared with the TQ-treated group, the survival area of flaps decreased in the TQ + EX527 group (Figures 6 A, B). Moreover, Doppler imaging revealed a decrease in blood flow intensity in the flaps of the TQ + EX527 group relative to the TQ-treated group (Figures 6 C, D). Histological analysis using HE staining showed a significant reduction in microvasculature in the TQ + EX527 group compared to the TQ-treated group (Figures 6 E, F). Additionally, WB assays demonstrated a significant downregulation of angiogenesis-related markers in the TQ + EX527 group (Figures 6 G, H). In summary, these findings indicate that the protective effects of TQ on flap survival are at least partially mediated through the SIRT1/NF-κB/NLRP3 pathway.

## Discussion

Perforator flaps are increasingly being utilized in clinical settings, yet local necrosis of perforator flaps has emerged as a significant issue caused by multiple factors. Angiogenesis is crucial for flap survival. In our study, we observed that TQ significantly increased microvascular density and CD34-positive endothelial cells in the flaps, thereby enhancing blood supply to rat perforator flaps. Furthermore, our results indicate that TQ upregulated the expression of cadherin-5, VEGF, and MMP9, raising the possibility that TQ promotes angiogenesis in rat perforator flaps and enhances flap survival by elevating these angiogenesis-related proteins. These findings suggest that TQ can improve the survival rate of perforator flaps after ischemia-reperfusion injury by facilitating angiogenesis.

Ischemia-reperfusion injury, characterized by the accumulation of ROS in ischemic tissues upon reperfusion and the subsequent induction of oxidative stress, is another factor contributing to flap necrosis (Kim and Hong, 2007). Superoxide dismutase (SOD) is a vital component of the cellular response to ROS-induced oxidative stres (Miao and St Clair, 2009). Similarly, the antioxidant effects of eNOS and HO1 are also present (Wang et al., 2013). In our study, we found that TQ treatment increased the protein expression levels of SOD1, HO1, and eNOS in rat perforator flap tissues, thereby conferring resistance to oxidative stress. Apoptosis, a normal form of programmed cell death, occurs in flap necrosis. Several studies have shown that TQ exerts anti-apoptotic effects by altering various cellular pathways (Bimonte et al., 2019). Our results demonstrate that TQ can prevent apoptosis in rat perforator flaps after ischemia-reperfusion, as indicated by the apoptosis-related proteins Bax, Bcl-2, and C-CASP3 mentioned above. In summary, our findings indicate that TQ protects perforator flaps from necrosis by reducing oxidative stress and apoptosis.

Pyroptosis, an emerging type of cell death, regulates critical physiological processes such as cell development, tissue homeostasis, stress responses, and inflammatory reactions (Robinson et al., 2019). Pyroptosis is often accompanied by ROS production and organelle damage (Pétrilli et al., 2007). Additionally, ROS upregulate the NF-κB signaling axis, which activates the NLRP3 inflammasome and ultimately triggers pyroptosis (Jia et al., 2019). Previous reports have shown that inhibiting pyroptosis can improve the viability of random flaps (Li et al., 2021a; Li et al., 2021b; Zhu et al., 2021). Prior studies have demonstrated that TQ can alleviate cardiac injury by inhibiting pyroptosis (Wang et al., 2022). To investigate the mechanisms underlying the role of TQ in rat perforator flap survival, we assessed its modulation of pyroptosis in rat perforator flaps after ischemia-reperfusion. In our current experiments, we found that TQ reduced the expression of NLRP3, GSDMD-N, Caspase-1, IL-1β, IL-18, and ASC proteins both *in vivo* and *in vitro*. Furthermore, the positive effects of TQ were inhibited when the SIRT1 inhibitor EX527 enhanced pyroptosis. All these findings suggest the potential role of TQ in improving flap survival by inhibiting the activation of pyroptosis.

Considering the pivotal roles SIRT1 plays in numerous diseases, stimulating SIRT1 may emerge as a therapeutic option to enhance the survival rate of skin flaps. Prior studies have demonstrated that SIRT1 is positively regulated in various conditions, including hypoxic-ischemic brain injury (Li et al., 2022), acute kidney injury (Lin et al., 2022), and acute lung injury (Fu et al., 2019). In this investigation, skin flap tissues treated with TQ exhibited a significant increase in SIRT1 protein expression. When SIRT1-specific inhibitor EX527 was utilized, the activating effect of TQ on SIRT1 protein was attenuated, leading to increased levels of pyroptosis, decreased angiogenesis, elevated oxidative stress and apoptosis, and ultimately, increased necrosis area in the skin flaps. Collectively, we hypothesize that TQ may limit pyroptosis in rat perforator flaps through SIRT1 modulation, thereby reducing flap necrosis.

Regarding inflammation, NF-κB serves as a major transcriptional regulator of inflammation-related genes (Leslie et al., 2013). Current research on random skin flap ischemia-reperfusion models confirms the significance of NF-κB in the pathophysiology of flap ischemia-reperfusion injury (Fan et al., 2021). In this study, TQ inhibited the phosphorylation of NF-κB following ischemia-reperfusion injury in rat perforator flaps, indicating its ability to suppress the NF-κB signaling pathway. It has been reported that SIRT1 inhibits the NF-κB pathway by directly deacetylating p65 at lysine 310 (Yeung et al., 2004). Notably, our study also suggests that the beneficial effects of TQ are partially mediated by SIRT1, as demonstrated by the effects of the SIRT1 inhibitor EX-527 mentioned above. With SIRT1 expression suppressed by EX-527, NF-κB phosphorylation and NLRP3-mediated pyroptosis-related proteins were significantly elevated. Typically, NF-κB is bound in the cytoplasm by its inhibitor IκB. Upon stimulation, IκB is degraded, allowing NF-κB to translocate to the nucleus and stimulate the expression of inflammatory genes. In this study, the SIRT1 inhibitor increased NF-κBp65 phosphorylation, indicating that NF-κB p65 acts as a downstream target of SIRT1. The acetylation of p65 represents a potential mechanism for the pro-inflammatory effects of SIRT1 inhibitors (Schug et al., 2010). Ultimately, our research indicates that TQ treatment can inhibit pyroptosis in perforator flaps through the SIRT1/NF-κB/NLRP3 signaling pathway, an effect that was reversed by the SIRT1 inhibitor EX-527.

## Summary

Our research demonstrates that TQ positively influences the survival of multi-regional perforator flaps primarily by inhibiting pyroptosis, promoting angiogenesis, and reducing apoptosis and oxidative stress accumulation. Additionally, *in vitro* experiments have shown that TQ can inhibit pyroptosis. Furthermore, we have discovered that SIRT1 plays a crucial role in mediating these processes within perforator flaps, suggesting it may serve as a potential therapeutic target for enhancing flap survival. By utilizing SIRT1 inhibitor EX527 and NLRP3 inhibitor MCC950, we have validated that the SIRT1/NF-κB/NLRP3 pathway may partially contribute to the beneficial effects of TQ on perforator flaps (Figure 6I).

## Data Availability

The datasets presented in this study can be found in online repositories. The names of the repository/repositories and accession number(s) can be found in the article/Supplementary Material.
