# Supplementary material for: Thymoquinone alleviates the accumulation of ROS and pyroptosis and promotes perforator skin flap survival through SIRT1/NF-κB pathway
Source: Front Pharmacol. 2025 Mar 25;16:1567762. doi: 10.3389/fphar.2025.1567762 (PMC11975933; doi:10.3389/fphar.2025.1567762)
Supplement: Supplementary file 1 [file Table1.docx]

| Protein | Compound | Binding Energy(kcal·mol^-1^) |
| --- | --- | --- |
| sirt1 | thymoquinone | -6.3 |
