# Supplementary figures and images for: Thymoquinone alleviates the accumulation of ROS and pyroptosis and promotes perforator skin flap survival through SIRT1/NF-κB pathway
Source: Front Pharmacol. 2025 Mar 25;16:1567762. doi: 10.3389/fphar.2025.1567762 (PMC11975933; doi:10.3389/fphar.2025.1567762)

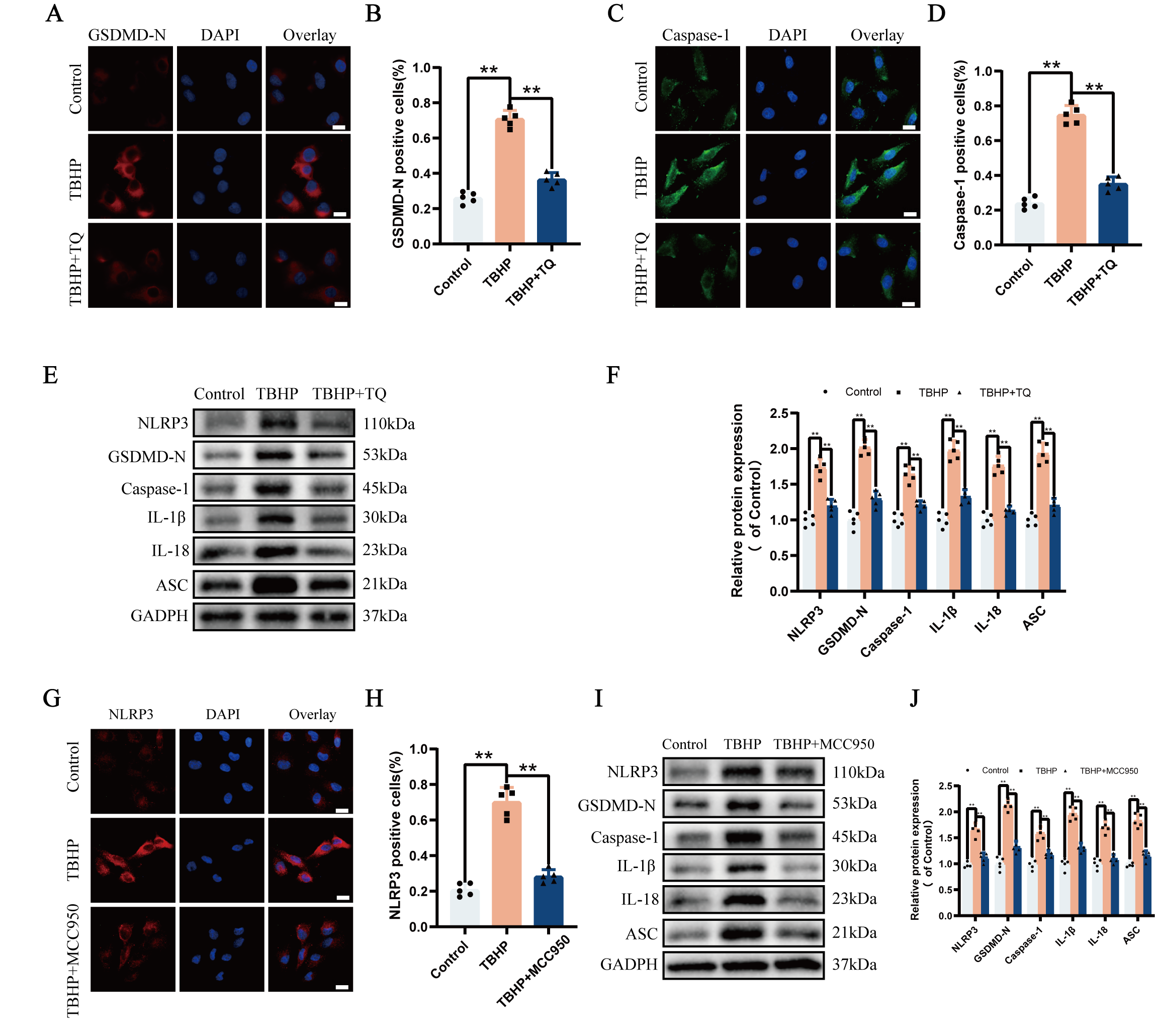

Supplement: Supplementary file 2 [file Image1.tif]
